# Aggressiveness and mycotoxin profile of *Fusarium avenaceum* isolates causing Fusarium seedling blight and Fusarium head blight in UK malting barley

**DOI:** 10.3389/fpls.2023.1121553

**Published:** 2023-03-08

**Authors:** Safieddin Inbaia, Arifa Farooqi, Rumiana V. Ray

**Affiliations:** Division of Plant and Crop Sciences, School of Biosciences, University of Nottingham, Loughborough, United Kingdom

**Keywords:** *Fusarium avenaceum*, Fusarium seedling blight, Fusarium head blight, enniatins, aggressiveness, malting barley

## Abstract

**Introduction:**

*Fusarium* avenaceum causing Fusarium seedling blight (FSB) and Fusarium head blight (FHB) on barley is associated with economic losses of crop yield and quality, and the accumulation of mycotoxins including the enniatins (ENNs) A, A1, B and B1. Although *F. avenaceum* is the main producer of ENNs, studies on the ability of isolates to cause severe Fusarium diseases or produce mycotoxins in barley are limited.

**Methods:**

In this work, we investigated the aggressiveness of nine isolates of *F. avenaceum* to two cultivars of malting barley, Moonshine and Quench, and defined their ENN mycotoxin profiles in *in vitro* and in planta experiments. We assessed and compared the severity of FSB and FHB caused by these isolates to disease severity by F. graminearum, *F. tricinctum and F. poae*. Quantitative real-time polymerase chain reaction and Liquid Chromatography Tandem Mass Spectrometry assays were used to quantify pathogen DNA and mycotoxin accumulation, respectively, in barley heads.

**Results:**

Isolates of *F. avenaceum* were equally aggressive to barley stems and heads and caused the most severe FSB symptoms resulting in up to 55% reductions of stem and root length. Fusarium graminearum caused the most severe FHB disease, followed by the isolates of *F. avenaceum* with the most aggressive *F. avenaceum* isolates capable of causing similar bleaching of barley heads as *F. avenaceum*. Fusarium avenaceum isolates produced ENN B as the predominant mycotoxin, followed by ENN B1 and A1 *in vitro*. However, only the most aggressive isolates produced ENN A1 in planta and none produced ENN A or beauvericin (BEA) either in planta or *in vitro*.

**Discussion:**

The capacity of *F. avenaceum* isolates to produce ENNs was related to the accumulation of pathogen DNA in barley heads, whilst FHB severity was related to the synthesis and accumulation of ENN A1 in planta. Cv. Moonshine was significantly more resistant than Quench to FSB or FHB, caused by any Fusarium isolate, and to the accumulation of pathogen DNA, ENNs or BEA. In conclusion, aggressive F. avenaceum isolates are potent ENN producers causing severe FSB and FHB with ENN A1 requiring further investigation as potential virulence factor for *F. avenaceum* in cereals.

## Introduction

Barley (*Hordeum vulgare* L.) is one of the oldest cultivated grain crops ranking fourth, among cereals, in importance for feed and food production around the world. Approximately 80%–90% of barley grain yield is destined for livestock feed, while the remaining 10% is converted into malt for brewing, distilling, and baking (Morcia et al., 2016; Food and Agriculture Organization (FAO, 2018).

Barley is susceptible to *Fusarium* spp., causing a complex of Fusarium diseases during crop development including seedling blight (FSB), foot rot, and Fusarium head blight (FHB) (Parry et al., 1995; Bai & Shaner, 2004; Ferrigo et al., 2016). Consecutive infections within the crop life cycle provide inoculum for each disease within the complex (Parry et al., 1995), with disease severity being a function of the aggressiveness of the causal pathogen towards the host at different growth stages, the cereal genotype, and the prevailing environmental conditions (Parry et al., 1995; Doohan et al., 2003). FHB of the Fusarium complex is considered the most economically damaging disease (Parry et al., 1995; Ferrigo et al., 2016), resulting in significant losses in crop yield and quality (Trail, 2009; Habler & Rychlik, 2016), and safety due to grain contamination with mycotoxins (Desjardins, 2006). The most common toxigenic causal organisms of FHB in barley include *F. avenaceum*, *F. poae* and *F. tricinctum* (Nielsen et al., 2014). *Fusarium avenaceum* and *F. tricinctum* produce predominantly moniliformin (MON) and enniatins (ENN A, A1, B and B1) (Kokkonen et al., 2010) and occasionally, beauvericin (BEA) (Logrieco et al., 2002). *Fusairum poae* produces nivalenol (NIV), diacetoxyscirpenol (Thrane et al., 2004) and BEA (Kokkonen et al., 2010). ENNs and BEA have similar toxicity and have been shown to induce apoptosis, increase cytoplasmic calcium concentration, and cause DNA fragmentation in mammalian cell lines (Bertero et al., 2020). MON has shown inhibitory action on several enzymes, including pyruvate dehydrogenase, α-ketoglutarate dehydrogenase, pyruvate decarboxylase and acetohydroxy acid synthase *in vitro* (Jestoi, 2008; Gruber-Dorninger et al., 2016; Mallebrera et al., 2018). ENNs, BEA and MON are collectively refered to as emerging mycotoxins (Jestoi et al., 2004) remaining under evaluation by the European Food Safety Authority (EFSA) Panel on Contaminants in Food Chain (CONTAM, 2014). Currently there are no legislative limits imposed on growers and processors for these mycotoxins and data is still being collected on their occurrence in cereals and toxicologial profiles. The most recent European surveys of Fusarium mycotoxins in barley have revealed that ENN B is most prevalent, followed by ENN B1, A1 and A (Bolechová et al., 2015; Habler & Rychlik, 2016; Jajić et al., 2019). Although *F. avenaceum* is the most common producer of ENNs (Gautier et al., 2020) studies on the aggressiveness and the ability of isolates to cause Fusarium diseases or produce mycotoxins in barley (Jestoi et al., 2008) are limited, whilst data specific to UK isolates is lacking. The last UK survey of more than 200 barley samples from England and Scotland over two consecutive years, reported that *F. avenaceum*, *F. poae* and *F. tricinctum* were present in 100%, 90% and 81% of the sampled grain, respectively, however ENNs or BEA were not quantified in these samples (Nielsen et al., 2014). The objectives of this work were to determine the aggressiveness of isolates of *F. avenaceum* isolated from these survey samples by assessing the ability of these isolates to cause Fusarium diseases and their capacity to produce emerging mycotoxins *in vitro* and in planta. The hypotheses of these studies were: i) *F. avenaceum* causes equally or more severe FSB or FHB disease on barley compared to isolates of other common *Fusarium* species; ii) isolates of *F. avenaceum* are potent producers of ENNs *in vitro* and in planta; and iii) mycotoxin production can be related to pathogen DNA and severity of FHB.

## Materials and methods

### Plant material

Series of *in vitro* and in planta experiments were conducted with two spring barley cultivars, Moonshine and Quench in 2017 and 2018 under glasshouse conditions at the University of Nottingham, Sutton Bonington Campus, UK (**Table 1**). Prior to being used in experiments, barley seeds were surface sterilised by immersion in 75% ethanol for 40 seconds followed by immersion in 0.5% sodium hypochlorite solution (NaOCl) for 1 minute. The seeds were then rinsed three times in sterile water and dried on a filter paper in a laminar flow cabinet.

**Table 1:** Isolates, inoculation methods and inoculated tissues used in Fusarium seedling blight (FSB) and Fusarium head blight (FHB) experiments performed with two barley cultivars, Moonshine and Quench.

Experiment	*Fusarium* species	ID isolate	Inoculation method	Inoculated tissue
FSB 1	*F. avenaceum*	40,75,219,225	Macerated mycelia in soil	Pre- or non-germinated seed
	*F. graminearum*	13,15,16		
	*F. tricinctum*	37,53,56		
	*F. poae*	9,175, 252		
FSB 2	*F. avenaceum*	40, 55, 74, 75, 210, 219, 225, 235, 248	Macerated mycelia in soil	Pre- or non-germinated seed
	*F. graminearum*	15		
	*F. tricinctum*	53		
	*F. poae*	175		
FHB	*F. avenaceum*	40, 55, 74, 75, 210, 219, 225, 235, 248	Conidial suspension 1×10^6^ ml^-1^	Heads at growth stage 59
	*F. graminearum*	15		
	*F. tricinctum*	53		
	*F. poae*	175		
*In vitro* mycotoxin production	*F. avenaceum*	40, 55, 74, 75, 210, 219, 225, 235, 248	Conidial suspension 1×10^6^ ml^-1^	Grain cv. Moonshine
	*F. tricinctum*	53		
	*F. poae*	175		

### Inoculum production

All *Fusarium* isolates, including *F. avenaceum*, *F. tricinctum*, *F. graminearum* shown in **Table 1** were isolated from naturally infected grain from the SAFEMalt project (Nielsen et al., 2014). Single spore isolates were stored short term on potato dextrose agar (PDA, Sigma-Aldrich, UK) at 20-25°C and long term in glycerol stocks at -80°C.

### FSB experiments

All experiments were repeated twice. Used isolates are shown in **Table 1**. The first experiments (FSB1) with each barley variety were designed as a randomised block with two factors, inoculation, and *Fusarium* isolate, with four replicates of each treatment combination. The purpose of this experiment was to compare *Fusarium* isolates of known identity, confirmed by real-time PCR, and pathogenicity isolated from naturally infected barley grain (Nielsen et al., 2014), and identify aggressive isolates of *F. graminearum*, *F. tricinctum* and *F. poae* to include as controls in subsequent experiments with larger numbers of isolates of *F. avenaceum*. The second series of glasshouse experiments (FSB2) were designed as a randomised block with nine isolates of *F. avenaceum* and the most aggressive isolates of *F. poae, F. tricinctum* and *F. graminearum* identified from FSB1 used as controls. Two barley varieties were included in the design as a factor with two levels, Moonshine or Quench.

Two different methods were used to determine the barley cultivar responses to FSB pre- and post-germination (Ren et al., 2015). For the former, barley seeds, without pre-germination, were sown directly in compost (John Innes No. 2) inoculated with five mycelial plugs from cultures grown on PDA at 20-25°C for 5–7 days. For the latter method, post-germination, surface-sterilised seeds were first incubated on filter paper saturated with sterile water at room temperature (22°C) for 2 days to obtain evenly germinated seeds.

To prepare inoculated soil, John Innes compost No. 2 was autoclaved for 1 hour at 121°C for two consecutive days and allowed to cool to room temperature before being used as a medium for growth in experiments. Plastic trays (Beekenkamp Verpakkingen, Netherlands) with 308 compartments (3.0 cm × 3.0 cm) were filled with compost and 5 ml of sterile water was added to each well. Soil in the tray wells was inoculated with five mycelial-agar plugs (5 mm diameter) of each isolate of included in the studies. To ensure that the inoculum was evenly distributed in the potting medium, the inoculated agar plugs were first macerated before being mixed well with the compost. Non-inoculated PDA agar plugs were used as control (mock-inoculated). Following soil inoculation, two pre-germinated or non -germinated seeds of each cultivar were sown in each well and then covered with a 2 cm layer of compost. The trays were incubated in a glasshouse at 10–18°C with a relative humidity of 70 ± 10% and a photoperiod of 9 hours using a combination of automatic vents and a heating system.

#### Visual disease of FSB and plant trait assessments

Barley seedlings were extracted from the compost at 15 days post-inoculation (dpi) and carefully washed with tap water to remove debris before visual disease and plant trait assessment. Symptoms of FSB were assessed visually on individual seedling stems by classifying the proportion of stem discoloration on a scale of 0–4 (0 = no lesions, clean seedling base; 1 = lesions affecting less than 25% of the base circumference; 2 = lesions affecting 26%–75% of the base circumference; 3 = lesions affecting more than 75% of the base circumference; 4 = dead; (Ren et al., 2015). Following disease assessment, root and stem length were measured using a ruler and recorded for individual seedlings.

### FHB experiments

FHB experiments were conducted in 2017 and 2018 at the University of Nottingham Sutton Bonington Campus in glasshouse conditions. The experimental designs for both experimental repeats were randomised blocks, with four replicates of each barley variety (cv. Moonshine or Quench) using the same *F. avenaceum* isolates and the most aggressive isolates of *F. poae*, *F. tricinctum* and *F. graminearum* used in the FSB experiments. The two barley varieties used in previous FSB experiments were grown into a 5 L pots at 10–18°C under a relative humidity of 70 ± 10% and a photoperiod of 9 h, using a combination of automatic vents and a heating system. The watering system delivered water directly to the compost twice a day. Barley plants were protected from powdery mildew using a Sulphur burner at weekly intervals, which was removed 3 weeks prior to head inoculation.

#### Fungal spore production and FHB inoculation of barley cultivars

*Fusarium avenaceum* isolates (**Table 1**) grown on PDA were incubated at 20°C for 14 days and exposed to near UV light for a period of 12 h every day to stimulate spore production for inoculation. Spores were harvested from culture plates by adding 5 ml of sterile distilled water, followed by agitation using a sterilised L-shaped plastic rod. The concentration of conidial suspensions was determined using a hemocytometer and adjusted to 1×10^6^ conidial ml^-1^ for each isolate. Spore solutions were used immediately for inoculation at anthesis of barley, growth stage (GS) 59 (Zadoks et al., 1974). Prior to inoculation, five ears of each plant were randomly labelled, and one droplet of spore suspension (total volume of 0.2 ml) was carefully applied, using syringe, on individual spikelets (between lemma and palea) of each labelled head of each plant. Control barley heads were mock-inoculated with sterile distilled water. Inoculated ears were covered with polyethene bags to achieve high humidity and stimulate FHB infection. The bags were removed 48 hours post-inoculation.

#### Visual FHB disease assessment

Visual disease assessments were carried out from mid-anthesis onwards (GS 65) at 7-days intervals over 28-day period. Inoculated heads (five per replicate) were assessed for numbers of spikelets showing water-soaked, necrotic lesions, or bleaching per total number of spikelets per head. The area under the disease progress curve (AUDPC) for each symptom (lesions or bleaching) was calculated using the mathematical formula by Shaner and Finney (1977), which is depicted below as an equation, where yi is the score of visually infected spikelets on the ith day, ti is the day of the ith observation and N is the total number of observations:


(Equation 1) $$AUDPC=\;\sum_{i=1}^{n-1}\left(\frac{yi+yi-1}{2}\right)(xi-x_{i-1})$$

#### Pathogen DNA quantification

Each inoculated head was individually harvested by hand at GS92 and threshed using a stationary thresher with care to retain *Fusarium*-damaged grains in the sample. Threshed grain was milled into a fine flour using a Krups F203 grinder (Krups, Windsor). Cross-contamination was avoided by thoroughly cleaning the grinding chamber between samples. Milled samples were stored at -20°C and consequently used for DNA extraction and quantification using the methods described by Nielsen et al. (2014).

Targeted pathogen DNA quantification was performed using real-time PCR assays with performed with CFX96 (Bio-Rad, UK). Ten-fold serial dilutions (1-10^-6^ ng µl^-1^) of DNA of *F. avenaceum* (isolate 75, University of Nottingham), *F. poae* (isolate 175, University of Nottingham) and *F. tricinctum* (isolate 53, University of Nottingham) were used to generate standard curves. PCR reactions consisted of a template of 2.5 µl DNA in a total reaction volume of 12.5 µl. The negative control used 2.5 µl of PCR-grade water in place of the DNA template. Species-specific primers were used for quantification of *F. avenaceum*, *F. tricinctum* and *F. poae* (**Supplementary Table 1**). 2x iQ SYBR Green Supermix (Bio-Rad, UK) and 250 nM of forward and reverse primers were used in PCR reactions. Linear regression was used to calculate the quantity of target pathogen DNA. Quantities of DNA were expressed as the amount of target DNA (in picograms) per total DNA in the sample (in nanograms). The limit of quantification was 10^-4^ pg ng^-1^ total fungal DNA, and all assays had an efficiency of 100.0%–104%. DNA of *F. graminearum* was not quantified by real-time PCR because this species did not produce the mycotoxins quantified in these studies which included BEA and ENNs.

### *In vitro* mycotoxin production by isolates of *F. avenaceum*

This experiment was performed twice and was set up as a completely randomised design with the isolates shown in **Table 1**. Barley grain of cv. Moonshine was soaked in sterile distilled water for 24 hours to increase the water content by approximately 50%, and the supernatants was discarded. Three replicates of soaked barley grains were weighed before adding 25 g of each replicate to a 100 ml conical flask, followed by autoclaving for 20 minutes at 121°C. The conical flasks were left to cool down for 3 hours and subsequently inoculated by adding 0.5 ml of 1 × 10^6^ ml^-1^ conidial spore suspension. Spore suspensions were prepared for each isolate by adding twelve mycelial plugs from actively growing culture to a conical flask (250 ml) containing carboxymethyl-cellulose (CMC) media. Flasks were placed on an orbital shaker for 6 days at room temperature. Spore solutions of each flask were separately filtered through two layers of sterile muslin cloth, quantified using a haemocytometer, then diluted in sterile water and adjusted to a concentration of 1×10^6^ spores ml^-1^. The spore suspensions were used immediately to inoculate the grain, and sterile distilled water (SDW) was added to the control samples. Inoculated barley was incubated at 22°C ± 1°C for 3 weeks. After the incubation period, inoculated grain from each flask was collected in a labelled paper bag. The samples were lyophilised and milled to a fine flour using a coffee grinder for each individual species of *Fusarium* to avoid cross-contamination. All samples were stored at -20°C until extraction and quantification of mycotoxins.

### Extraction and quantification of mycotoxins using LC-MS/MS

Organic solvents used for mycotoxin extraction and LC-MS/MS analysis (HPLC grade acetonitrile and methanol) were purchased from Sigma-Aldrich, Germany. Certified standards (purity of 99%) of BEA, ENNs A, A1, B and B1 were purchased from Alexis Biochemicals, New York. All solvents were filtered through a cellulose filter of 0.2 µm before use. Individual stock solutions of 1 mg ml^-1^ of BEA and ENNs A, A1, B and B1 standards were prepared using acetonitrile (ACN HPLC grade). Mixed mycotoxin standard (BEA and ENNs A, A1, B and B1) with a concentration of 10 µg ml^-1^ was used for the preparation of standard curves of mycotoxins with 4-fold series dilution ranging between 4-0.0039 µg ml^-1^. Linearity for all toxin standard curves was determined as R^2 =^ 0.98-0.99. Calibration standards were used for quantification by spiking the matrix with external standards prior to extraction. The extraction method for BEA and ENNs (A, A1, B and B1) used a mixed solvent consisting of 10 ml of 0.1% formic acid HCOOH and 10 ml ACN added to 5 g of a milled grain sample in a 50 ml Falcon bottle. The mixture was shaken in the FastPrep 5G system (MPBio, USA) for 2 minutes, and 1 g of NaCl and 4 g of MgSO_4_ were added to the mixture and centrifuged at 5000 rpm for 5 minutes. Without disturbing the pellet, 1 ml of the supernatant was transferred to a clean HPLC vial. The extracted sample was subsequently filtered through a 0.2 µm nylon micro-filter. Limits of detection (LOD), quantification (LOQ) and recovery were determined by spiking the matrix with mycotoxin solutions at 50, 100 and 200 µg ml^-1^. LOD and LOQ for BEA were 0.3 µg ml^-1^ and 3 µg ml^-1^, respectively. LOD and LOQ for ENN B, B1, A1 and A were 0.6 and 2, 0.8 and 4, 0.1 and 2, and 0.4 and 3 µg ml^-1^, respectively. Recovery for all toxins ranged between 86 and 99%.

LC-MS/MS analysis was performed on an Agilent 1200 Infinity LC system (Agilent Technologies, Germany) with a binary pump, coupled with the Agilent 6490 MS/MS ESI. The chromatographic separation of BEA and ENNs was conducted at 24 ± 1°C on a reverse phase C18 column OOG-4252-40 (5 µm 250 × 3.0 mm). The mobile phase consisted of 0.1% (v/v) formic acid and 1 mM ammonium formate in methanol. An isocratic pump system was used to provide a mobile phase with a flow rate of 0.6 ml min^-1^. To avoid the formation of any gas bubbles, the column was washed with mobile phase for 20 minutes at different flow rates of 2 ml min^-1^ for 5 minutes, then 1 ml min^-1^ for 10 minutes before reverting to the requested flow rate of 0.6 ml min^-1^, which was maintained for 5 minutes. The injection volume was 10 μl and the total run time was 5 minutes. The used ESI interface was in positive ion mode with a source temperature of 100°C, desolvation temperature of 450°C, cone nitrogen gas flow of 60 L/h, desolvation gas flow of 450 L/h and capillary voltage of 3.5 kV. BEA and ENNs A, A1, B and B1 were analysed in MRM. The precursor and fragment ions for each toxin are shown in (**Supplementary Table 2**). The resolution for the first and third quadruples was set to 10.0 (unit resolution). Chromatograms were processed using MassLynx (v3.2) to integrate and quantify the peak areas of detected mycotoxins in both the standards and the extracted samples. The peak areas for BEA; ENNs A, A1, B and B1 in the samples were confirmed by comparing the retention time of the peak area with those of standard solutions, as well as by recognizing both the precursor and product ions and their ratio (**Supplementary Table 2**). The linearity for each toxin was evaluated using standard solutions in a pure solvent and matrix-matched calibration curves. BEA and ENN concentrations in samples were calculated based on the plotted external standard calibration curves.

### Statistical analysis

All data was analysed using Genstat Version 14.1 for Windows. A *p*-value of less or equal to 0.05 was considered statistically significant. All data was analysed using analysis of variance (ANOVA) with experimental repeats included as a factor in the treatment structure with up to 4-way significant interactions between factors tested. Where there were no interactions between factors and experiment, experiments were used as replicates in ANOVA. Prior to ANOVA residuals were checked for normal distribution and where needed data were log_10_ transformed to obtain normal distribution. Single linear regression (SLR), multiple linear regression (MLR) or non-linear standard curve fitting with groups for cultivar were performed to determine the relationships between visual disease symptoms, pathogen DNA and mycotoxins, with data for the two cultivars being tested for position and parallelism using full data sets for experimental repeats.

## Results

### Fusarium seedling blight disease severity caused by UK isolates of *F. avenaceum*

**Figure 1:**
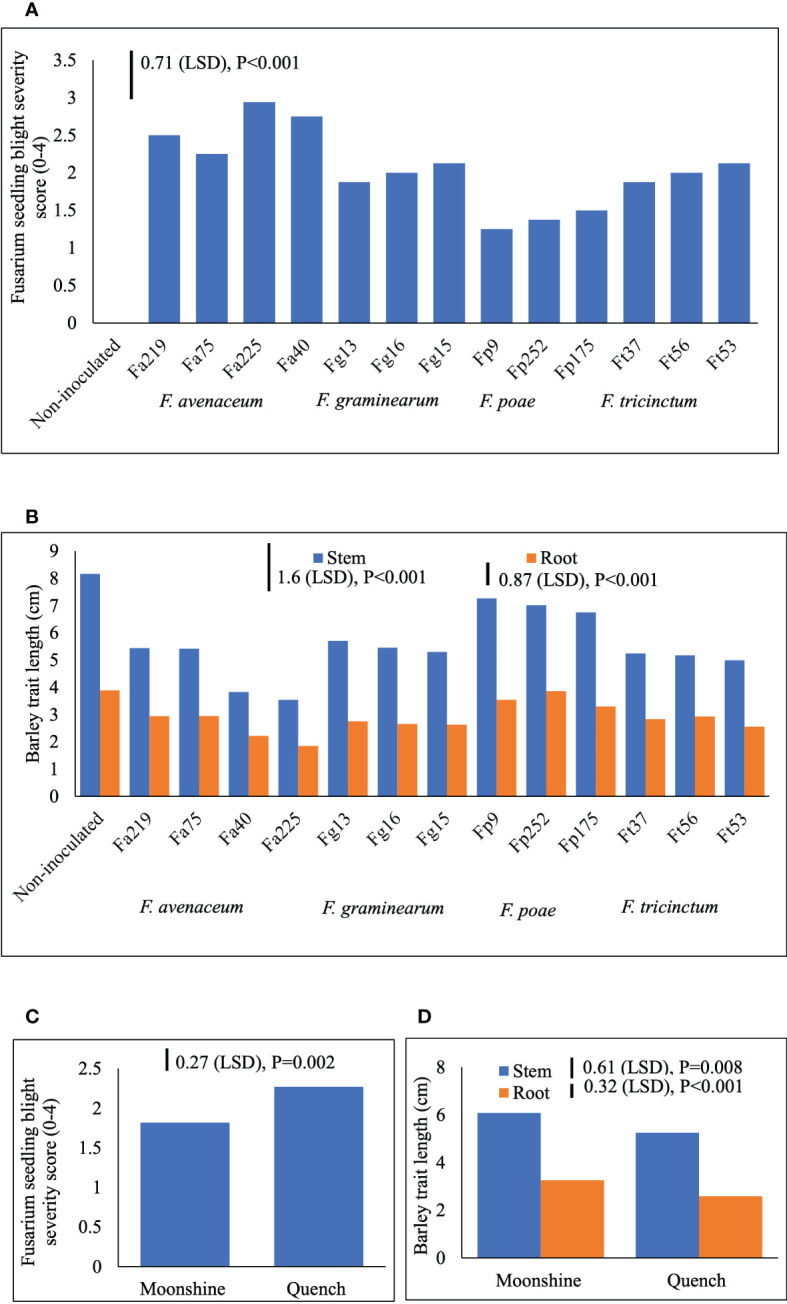
Severity of Fusarium seedling blight (FSB) by isolates of *F avenaceum*, *F poae*, *F tricinctum* and *F graminearum* **(A)**, and effects of soil-borne disease on stem and root length (cm) **(B)** of barley (cvs. Moonshine and Quench) **(C, D)**. *Fusarium avenaceum* (Fa); *F graminearum* (Fg); *F poae* (Fp); *F tricinctum* (Ft); FSB was assessed visually on seedlings by classifying the proportion of stem discoloration based on a 0–4 scale (0=no lesions, clean seedling base; 1=lesions affecting less than 25% of the base circumference; 2=lesions affecting 26–75% of the base circumference; 3=lesions affecting more than 75% of the base circumference; 4= dead). Analysis of variance includes two experimental replicates, LSD=least significant difference with individual p values shown.

**Figure 2:**
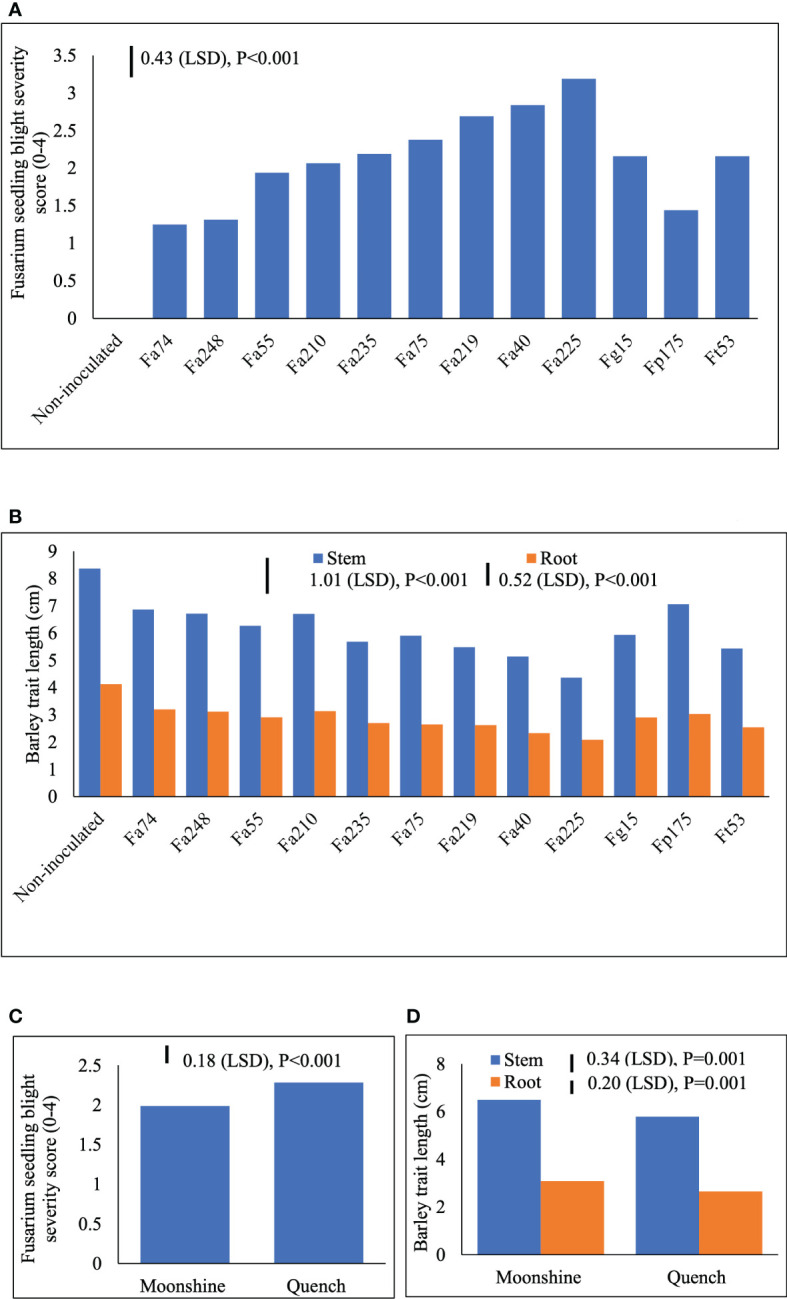
Severity of Fusarium seedling blight (FSB) by nine isolates of *F avenaceum* and single isolates of *F poae*, *F tricinctum* and *F graminearum* (included as controls) **(A)** and effects of soil-borne disease on stem and root length (cm) **(B)** of barley (cvs. Moonshine and Quench) **(C, D)**. *Fusarium avenaceum* (Fa); *F graminearum* (Fg); *F poae* (Fp); *F tricinctum* (Ft); FSB was assessed visually on seedlings by classifying the proportion of stem discoloration based on a 0–4 scale (0=no lesions, clean seedling base; 1=lesions affecting less than 25% of the base circumference; 2=lesions affecting 26–75% of the base circumference; 3=lesions affecting more than 75% of the base circumference; 4= dead). Analysis of variance includes two experimental replicates, LSD=least significant difference with individual p values shown.

Two different FSB (FSB1 and FSB2) experiments were carried out to determine cultivar responses to pre- or post-germination seedling blight caused by isolates of *F. avenaceum*, *F. graminearum*, *F. tricinctum* and *F. poae*, and the effect of FSB on seedling traits of barley. To address the first objective non-germinated and pre-germinated seeds were used in both experiments. Results showed that there were no significant differences between methods for seed inoculation (pre- or post-germination) or interactions with any other experimental factors for FSB severity or effects of disease on stem or root length (*p*>0.05). Similarly, there were no significant interactions for cultivar or isolate in either of the FSB experiments (*p*>0.05). Infection by isolates of *F. avenaceum*, *F. graminearum* and *F. tricinctum* resulted in similar disease symptoms of dark brown, elongated lesions on the stems of barley seedlings, whist *F. poae* caused milder symptoms. There were significant differences in the aggressiveness of isolates belonging to different *Fusarium* species (**Figure 1A**). Isolates *F. avenaceum* caused the most severe FSB, followed by *F. graminearum*, *F. tricinctum* and *F. poae* (**Figure 1A**). In both varieties, Fa225 caused lesions affecting more than 75% of the stem circumference while Fp9 was the least pathogenic isolate to barley stems. Of all isolates of *F. graminearum*, *F. tricinctum* and *F. poae* included in these studies, FSB symptoms by Fg15, Ft53 and Fp175 were most severe and these isolates were thus chosen as controls for the subsequent experiments inclusive of more isolates of *F. avenaceum* (**Figure 2A**). A significant variation in the aggressiveness of *F. avenaceum* isolates (*p*< 0.001) causing FSB disease in barley was observed in FSB 2 experiments (**Figure 2A**), with Fa225 consistently causing the most severe symptoms (disease score of 3.25) in cv. Quench. In contrast, Fa74 was the least aggressive isolate with disease score of 1.00 in cv. Moonshine (**Figure 2A**). *F. graminearum* (Fg15) and *F. tricinctum* (Ft53) caused FSB of equal, and moderate, severity (score 2.16) and were both more aggressive to stems than *F. poae* (Fp175) causing only slight symptoms (score 1.44) (**Figure 2A**). FSB by isolates of *F. avenaceum, F. graminearum* and *F. tricinctum* reduced root and stem length of the two barley cultivars (**Figures 1B**, **2B**). In both experiments, cv. Quench was significantly more susceptible to pre- or post-germination FSB caused by any of the *Fusarium* isolates (**Figures 1C**, **2C**). Reductions of stem and root length were 20% greater in cv. Quench than in cv. Moonshine (**Figures 1D**, **2D**).

### Mycotoxin production by isolates of *F. avenaceum*

There were significant differences (*p*< 0.001) in the capacity of individual isolates of *F. avenaceum* to produce specific toxins (**Table 2**). *F. avenaceum* isolates synthesised the highest concentrations of ENN B followed by B1 and A1, whilst BEA and ENN A were not detected. The concentrations of ENN A1, B and B1 produced by *F. avenaceum* isolates *in vitro* ranged between 24.21- 1150.80, 2013.72-81,283.05 and 776.25-7533.56 µg kg^-1^, respectively (**Table 2**). Fa225 and Fa40 along with Fa75 and Fa219 were the most potent producers of ENNs B, B1 and A1, while Fa74 produced significantly lower amounts of ENNs B, B1 and less A1 compared to the other isolates. The single isolate of *F. tricinctum* Ft53 produced ENNs A1, B and B1 with a mean concentration of 25.35, 38904.51 and 709.58 µg kg^-1^, respectively. Fp175 produced only BEA with a concentration of 2330 µg kg^-1^.

**Table 2:** Concentrations of Enniatins A1, B, B1 produced by isolates of *F. avenaceum* and single isolate of *F. tricinctum* (included as control) *in vitro* on grain of cv.

Fusarium isolates	Mycotoxin Concentrations Log_10_ (µg kg^-1^)		
	ENN A1	ENN B	ENN B1
Fa210	1.80 (62.81)	3.96 (9036.49)	3.01 (1013.91)
Fa219	3.01 (1013.91)	4.73 (53333.49)	3.84 (6870.68)
Fa225	3.06 (1150.80)	4.91 (81283.05)	3.88 (7533.56)
Fa235	2.15 (139.96)	4.37 (23280.91)	2.96 (912.01)
Fa248	1.84 (69.02)	4.09 (12189.90)	2.83 (674.53)
Fa40	3.01 (1020.94)	4.79 (61659.50)	3.89 (7816.28)
Fa55	2.06 (114.82)	4.41 (25527.01)	3.52 (3296.10)
Fa74	1.38 (24.21)	3.30 (2013.72)	2.89 (776.25)
Fa75	2.92 (829.85)	4.76 (57942.87)	3.58 (3784.43)
Ft53	1.40 (25.35)	4.59 (38904.51)	2.85 (709.58)
P-value	<0.001	<0.001	<0.001
LSD	0.63	0.68	0.83
CV%	9.3	13.4	3.0

a back-transformed means shown in parentheses.

Moonshine.

### Fusarium head blight disease severity caused by isolates of *F. avenaceum*

**Figure 3:**
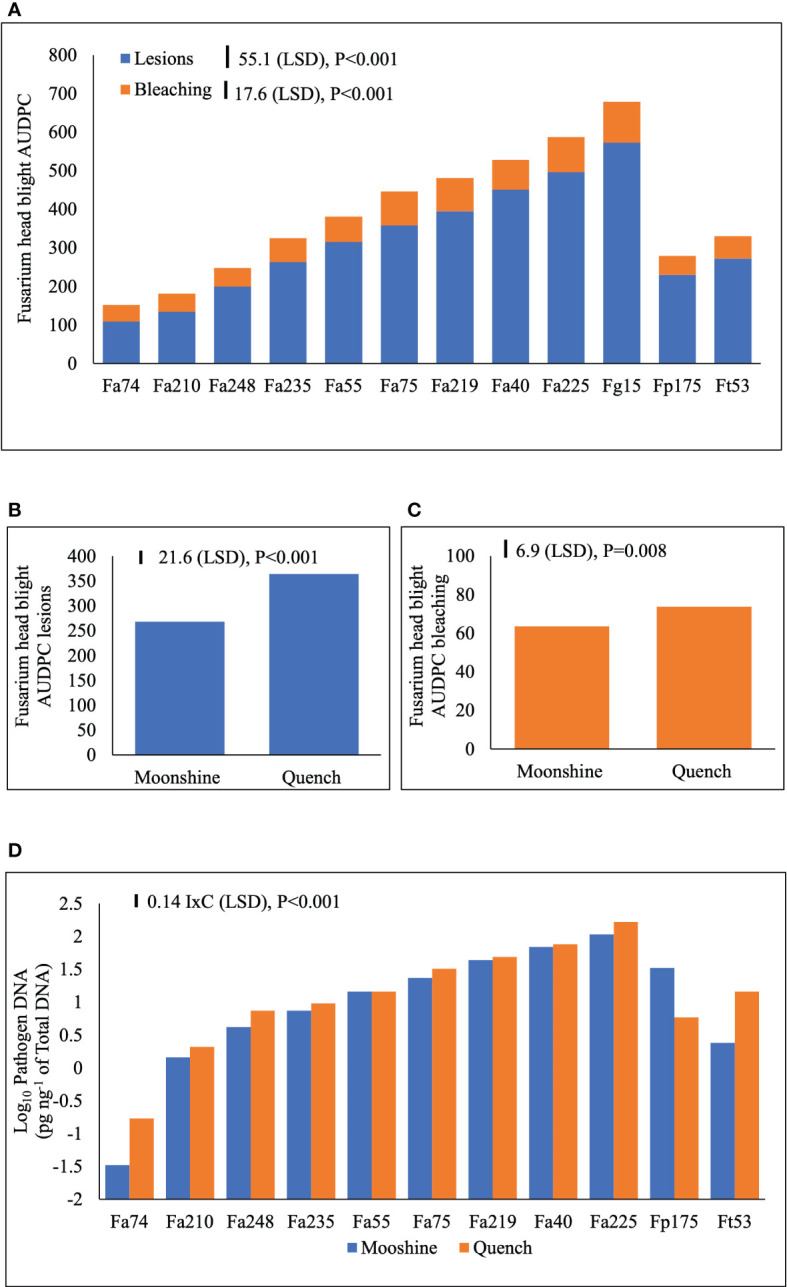
Area Under Disease Progress Curve (AUDPC) for Fusarium head blight (FHB) lesions and bleaching symptoms **(A)** on barley (cvs. Moonshine and Quench) **(B, C)** and pathogen DNA (pg ng^-1^ of total DNA) of *F. avenaceum* isolates and one isolate of *F. graminearum*, *F. poae* and *F. tricinctum* (included as controls) **(D)**. AUDPC calculated for the appearance of symptoms of lesions and bleaching due to FHB on barley heads for a period of 28 days following inoculation. DNA data is Log10 transformed prior to the analysis of variance inclusive of two experimental replicates. LSD=least significant difference with individual p values shown. I=isolate, C=cultivar. There were no observed disease symptoms or quantifiable pathogen DNA in the non-inoculated control, which was therefore excluded from the analysis. DNA of *F. graminearum* was not quantified as this species does not produce enniatins or beauvericin. Analysis includes two experimental replicates.

FHB disease symptoms are presented as AUDPC for lesions and bleaching (**Figure 3A**). There were no significant interactions (*p*>0.05) between the main treatment factors and individual experiments and therefore experiments were used as replicates in ANOVA. There were no significant interactions (*p*>0.05) between isolates and cultivars in the two experiments indicating consistency of the main effects. FHB lesions (**Figure 3B**) and bleaching (**Figure 3C**) developed more severely in cv. Quench than in cv. Moonshine (*p*< 0.001). There were distinct differences between isolates in their ability to cause FHB lesions or bleaching symptoms. Overall, the control *F. graminearum* isolate, Fg15, caused significantly more lesions over time compared to any of the isolates of *F. avenaceum* or the single control isolates of *F. tricinctum* or *F. poae* (**Figure 3A**). However, Fa225 was equally aggressive as Fg15 in causing spikelet bleaching and overall, these two isolates accumulated significantly greater AUDPC for bleaching compared to the rest (**Figure 3A**). The single isolates of Fp175 and Ft53 caused moderate FHB symptoms and were comparable to number of slight to moderately aggressive *F. avenaceum* isolates (**Figure 3A**). Of all isolates tested Fa74 was the least virulent strain to barley heads, consistent with the least DNA accumulation in tissues at the end of the experiments (**Figure 3D**). Significant interactions (*p*< 0.001) between isolates and cultivars were detected for pathogen DNA amounts of isolates of *F. avenaceum*, *F. tricinctum* and *F. poae* (**Figure 3D**). All *F. avenaceum* isolates and the single control isolate of *F. tricinctum* accumulated greater amounts of DNA in the more susceptible cultivar Quench, although DNA concentrations of Fa219, 40, 55 and 75 were not significantly different between cultivars. However, the single isolate of *F. poae* accumulated significantly more DNA in cv. Moonshine than in cv. Quench compared to *F. tricinctum* and *F. avenaceum* isolates Fa74, Fa210 and Fa248. Notably, the DNA of *F. graminearum* was not quantified by real-time PCR because this species does not produce any emerging mycotoxins which were the focus of these studies.

### Mycotoxin production in planta

The predominant mycotoxin produced by isolates of *F. avenaceum* in barley heads was ENN B followed by ENNs B1 and A1 (**Table 3**). None of the isolates produced BEA or ENN A and not all isolates produced ENN A1 in planta. BEA of significantly higher concentration (977.23 µg kg^-1^) in cv. Quench than in cv. Moonshine was only produced by the isolate of *F. poae*. There were significant interactions between isolates and cultivars for ENN A1 and ENN B1 as the single isolate of *F. tricinctum* and Fa40 produced similar concentrations of these mycotoxins in both cultivars (**Table 3**). In contrast, all other isolates produced significantly greater amounts of ENNs in cv. Quench compared to cv. Moonshine. There was significant variation (*p*< 0.001) between *F. avenaceum* isolates for their ability to produce ENN B. Fa225 produced the highest amount of ENN B (7512 µg kg^-1^) in cv. Quench, whilst the lowest concentration of ENN B was quantified as 699 µg kg^-1^, produced by Fa74, in cv. Moonshine. Cv. Moonshine accumulated less ENNs compared to cv. Quench and this effect was consistent for all isolates producing ENN B (**Table 3**). The single isolate of *F. tricinctum* Ft53 produced ENNs A1, B and B1, with the higher concentrations of 343.6, 4876.4 and 2717.7 µg kg^-1^, respectively, in cv. Quench.

**Table 3:** Concentrations of Enniatins A1, B, B1 and Beauvericin (µg kg^-1^) produced by *F. avenaceum* isolates and one isolate of *F. tricinctum* and *F.poae* included as controls on barley (cvs. Moonshine and Quench)

Fusarium isolates	Enniatin A1		Enniatin B		Enniatin B1		Beauvericin	
	Moonshine	Quench	Moonshine	Quench	Moonshine	Quench	Moonshine	Quench
Fa210	ND	ND	2.91(814.7)	3.21(1603.6)	2.64(435.3)	2.77(590.2)	ND	ND
Fa219	2.27(185.8)	2.56(359.7)	3.53(3402.5)	3.64(4387.3)	3.19(1546.7)	3.48(3017.2)	ND	ND
Fa225	2.79(609.5)	3.03(1074.0)	3.74(5495.4)	3.88(7512.8)	3.57(3683.8)	3.71(5094.5)	ND	ND
Fa235	ND	ND	3.42(2634.5)	3.58(3787.0)	2.90(786.5)	3.14(1376.6)	ND	ND
Fa248	ND	ND	3.20(1594.4)	3.40(2529.3)	2.88(766.7)	3.12(1326.5)	ND	ND
Fa40	2.76(576.8)	2.98(957.2)	3.66(4536.3)	3.83(6697.3)	3.60(3954.6)	3.66(4536.3)	ND	ND
Fa55	ND	ND	3.42(2610.4)	3.58(3772.2)	3.12(1312.8)	3.25(1779.5)	ND	ND
Fa74	ND	ND	2.84(699.0)	3.12(1310.7)	2.59(392.0)	2.77(586.3)	ND	ND
Fa75	2.19(156.3)	2.54(343.6)	3.47(2960.1)	3.69(4876.4)	3.27(1847.1)	3.43(2717.7)	ND	ND
Ft53	1.97(92.3)	2.05(111.2)	3.38(2386.7)	3.53(3399.4)	2.90(801.5)	3.01(1020.9)	ND	ND
Fp175	ND	ND	ND	ND	ND	ND	2.21(162.2)	2.99(977.2)
	P-value LSD	CV	P-value LSD	CV	P-value LSD	CV	P-value LSD	CV
Isolate	<0.001 0.07	4.1	<0.001 0.08	3.5	<0.001 0.05	2.2	--	–
Cultivar	<0.001 0.04		<0.001 0.03		<0.001 0.02		<0.001 0.02	6.5
Isolate*Cultivar	0.009 0.10		0.466 0.11		<0.001 0.07		--	–

a Back transformed means shown in parentheses. Analysis includes two experimental replicates. Fusarium avenaceum (Fa); F. tricinctum (Ft); F. poae (Fp). ND – not detected.

### Relationships between FHB AUDPC, pathogen DNA and enniatin concentrations

**Figure 4:**
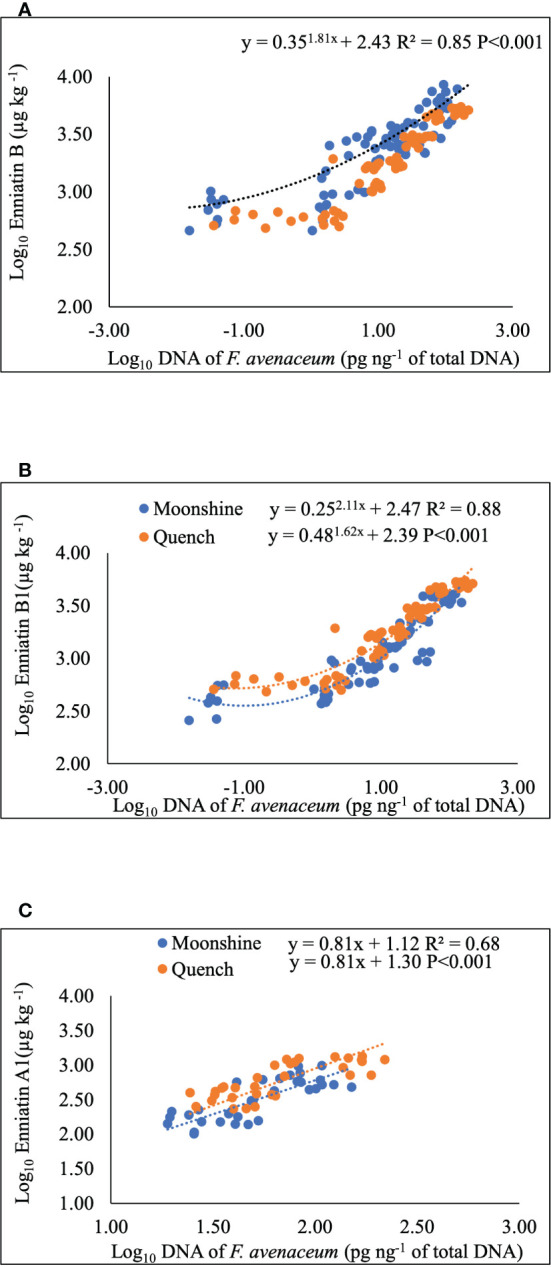
Non-linear, exponential curves for Enniatin B **(A)** and B1 **(B)** and single linear regression for Enniatin A1 **(C)** and *F avenaceum* DNA. Data for cvs. Moonshine and Quench fitted common line **(A)**, separate lines **(B)** and parallel lines **(C)**. Enniatins (µg kg^-1^) and DNA (pg of *F avenaceum* DNA per ng of total DNA) and Log10 transformed. Analysis using cultivar as a group including all data from two experimental replicates, equations and p values for position and parallelism are shown.

Regression analysis was performed to determine any relationships between visual disease symptoms, pathogen DNA and mycotoxin accumulation in barley heads (**Figure 4** and **Table 4**). DNA of *F. avenaceum* explained 63% of the variation in FHB AUDPC for lesions (*p*< 0.001), with data for the two barley cultivars fitting separate lines with different slopes and intercepts for each cultivar (**Table 4**). ENN A1 was also positively related to AUDPC for lesions, however, the relationship was of a moderate strength (R^2 =^ 0.37, *p*< 0.001) (**Table 4**). MLR revealed significant relationship between FHB AUDPC for bleaching and two of the enniatins, ENN A1 and ENN B, with the data fitting parallel lines with the same slope but different intercepts for each cultivar (**Table 4**). Strong (R^2 =^ 0.68-0.88, *p*< 0.001), positive relationships were also found, using non-linear and linear regression analysis, between DNA of *F. avenaceum* and individual ENNs. Mycotoxin and DNA data for the two barley cultivars fitted common exponential curve for ENN B (**Figure 4A**), separate exponential curves for ENN B1 and parallel linear lines for ENN A1 for the cultivars used in these studies.

**Table 4:** Relationships between FHB area under the disease progress curve (AUDPC) for lesions and bleaching by *F. avenaceum*, log_10_ ENN A1, ENN B, ENN B1 (µg kg^-1^) and log_10_ DNA (pg of *F. avenaceum* DNA per ng of total DNA) or cvs. Moonshine and Quench.

Response variate		Explanatory variate	Regression equation	R^2^	P-value
FHB AUDPC lesions FHB AUDPC bleaching		Log_10_ DNA Log_10_ ENN A1 Log_10_ DNA Log_10_ ENN A1(x)+ENN B (x_1_)	Moonshine, Y=92.9x+169.1; Quench, Y=157.6x+177.9 Moonshine, Y=178.9x -91; Quench, Y=178.9x-3 Moonshine, Y=16.5x+48.7; Quench, Y= 27.64x+53.7 Moonshine, Y=28.6x+36.8x_1_-119.4; Quench, Y=28.6x+36.8x_1_-103	0.63 0.37 0.44 0.37	<0.001 <0.001 <0.001 <0.001

## Discussion

Results from these studies showed that soil-borne *F. avenaceum* caused the most severe pre- and post-germination FSB disease in barley compared to *F. graminearum* and *F. tricinctum*. In contrast, isolates of *F. poae* were at best weakly pathogenic to seedlings and caused only slight disease symptoms agreeing with results from Imathiu et al. (2010), who reported that *F. poae* failed to produce seedling blight disease not just in wheat but also in oats. FSB can be initiated by seed or soil-borne inoculum, and previous studies have shown that soil-borne *F. graminearum* is less effective in causing damaging FSB in wheat compared to other species such as *Microdochium nivale* (Brown et al., 2021). Although *F. avenaceum* at species level was most aggressive to barley seedlings, there was significant variation between isolates with Fa225 causing the most, and Fa74 the least, severe symptoms in barley seedlings. Reductions of up to 55% in stem or root length by the most aggressive isolate (Fa225) showed that pre- or post-germination FSB causes severe inhibition of growth of barley seedlings. Growth reduction in wheat seedlings has been previously directly related to concentrations of ENNs B and B1 with root elongation being most inhibited (Burmeister and Plattner, 1987). Indeed, isolates of *F. avenaceum* included here produced considerable amounts of ENN B, B1 and A1. Similar toxigenic profile of *F. avenaceum* has been reported in several studies (Langseth, 1998; Logrieco et al., 2002; Jestoi et al., 2008; Kokkonen et al., 2010; Yli-Mattila et al., 2022), however, these isolates of *F. avenaceum* from UK barley grain were not able to produce ENN A or BEA. Although ENNs and BEA producing *Fusarium* spp. share common metabolic pathway enabled by the multienzyme enniatin synthase (ESYN1) encoded by *esyn1* (Urbaniak et al., 2020), BEA is rarely produced by *F. avenaceum* strains (Yli-Mattila et al., 2022) and in our studies was only produced by the isolate of *F. poae*. *Fusarium poae* is one the most common producers of BEA in cereals (Thrane et al., 2004; Mallebrera et al., 2018), and the overall mycotoxin accumulation as with any Fusarium mycotoxins can be affected by environmental conditions such as water content and temperature (Kokkonen et al., 2010). The isolate of *F. tricinctum* had a similar mycotoxin profile to *F. avenaceum* which was expected since both species are members of the *F. tricinctum* species complex in durum wheat and barley with *F. avenaceum* predominating (47.9%) in the complex (Senatore et al., 2021). In our studies, the single isolates of *F. tricinctum* and *F. poae* were included as controls for disease phenotyping and to confirm the mycotoxin profile at species level more isolates should be tested. However, from the three species, *F. avenaceum*, *F. tricinctum* and *F. poae* that predominate in recent surveys of cereal crops, only the first two species are implicated in significant accumulation of ENNs in grain (Orlando et al., 2019).

Pereyra and Dill-Macky (2010) reported that *F. avenaceum* is the second most aggressive species after *F. graminearum* to barley and wheat heads. Similarly, in our studies the control *F. graminearum* isolate (Fg15) caused the most severe FHB disease symptoms in both barley cultivars. However, all isolates of *F. graminearum* included in our studies were assessed as less aggressive to seedlings compared to the isolates of *F. avenaceum*, suggesting a variation in the pathogenicity of *F. graminearum* (Fg15) to different tissues of the barley plant. In contrast, the isolates of *F. avenaceum* demonstrated equal aggressiveness as FSB or FHB pathogens. Thus, the same isolates which caused severe FSB also caused the most severe FHB. Of all *F. avenaceum* isolates tested here Fa225, Fa40, Fa219 and Fa75 were found to be the most aggressive FSB and FHB pathogens. These isolates were also the most potent producers of ENN A1, B and B1 in *in vitro* and in planta experiments. Isolates (Fa235 and Fa55) which were assessed as moderately aggressive or weakly pathogenic (Fa248, Fa210 and Fa74) to stems or heads caused less severe FSB or FHB, respectively, consistent with lower production of ENN A1, B and B1 *in vitro* and absence of ENN A1 in planta. These results clearly suggest a strong association between the capacity of *F. avenaceum* isolates to produce ENNs but more importantly to specifically synthesise ENN A1 in planta for increased severity of FHB disease. Furthermore, linear regression analysis showed significant relationships between ENN A1 and FHB AUDPC for lesions, whilst 37% of variation in AUDPC for bleaching was explained by both ENN A1 and ENN B. Interestingly, ENN B1 was not a significant variate in any of the models and was excluded from the linear regressions. Similar, stronger, positive relationships were observed between pathogen DNA and AUDPC or ENN production, suggesting that fungal growth in planta is necessary for successful disease progression and mycotoxin accumulation. The role of ENNs in virulence of *F. avenaceum* is controversial since in some crops such as potatoes there is published evidence that ENN production increases necrotic lesion size (Eranthodi et al., 2020). However, in cereals, there is no conclusive evidence provided, and this maybe because certain quantity and quality of ENNs is required for increased virulence on specific crops, or perhaps ENNs act synergistically with other toxic fungal factors to provide support for increased virulence. In any case, this is the first report to show strong correlative evidence that the presence and increased quantity of ENN A1 contributes to aggressive *F. avenaceum* - barley interactions. Therefore, the role of ENN A1 in virulence of *F. avenaceum* to cereals should be investigated further.

The most significant factor which influenced the severity of FSB and FHB, and mycotoxin accumulation was the barley cultivar. Cv. Moonshine was significantly more resistant, than Quench, to FSB or FHB by any *Fusarium* isolate and to ENN and BEA accumulation. This agrees with previous results suggesting consistent resistance responses to more than one disease in the Fusarium complex in some cereal genotypes (Ren et al., 2015). Differences between genotypes used here were supported by results of the linear regression analysis demonstrating increased accumulation of ENNs for the same amount of pathogen DNA in Quench, compared to Moonshine. Furthermore, FHB lesions and bleaching developed more rapidly over time and were more severe per unit of *F. avenaceum* DNA in Quench. Further studies to evaluate cv. Moonshine within larger panel of barley genotypes of known resistance phenotypes, exposed to diverse number of *Fusarium* species will help to determine if this resistance can be utilised for improved control of Fusarium diseases.

At present, ENNs are neither routinely determined nor legislatively regulated (Jajić et al., 2019), and a limited range of data is available on their toxicity and occurrence in different cereals (Polišenská et al., 2020). The results from the experiments here showed that *F. avenaceum* isolates are aggressive FSB and FHB pathogens of barley and potent producers of ENNs that can contaminate barley grain in field. Therefore, more research attention is needed on this *Fusarium* pathogen present in many different geographical environments and predominating in barley. Further research is needed to investigate the potential variability in the aggressiveness of *F. avenaceum* and yield reduction, as well as the role of distinct metabolites including moniliformin in aggressiveness and virulence to cereals.

## Data availability statement

The raw data supporting the conclusions of this article will be made available by the authors, without undue reservation.

## Author contributions

SI and RR contributed to conception and design of the study. SI performed the experimental work, SI and RR performed the statistical analysis. SI and AF wrote the first draft of the manuscript. RR wrote sections of and edited the manuscript. All authors contributed to manuscript revision, read, and approved the submitted version.
