# Supplementary material for: Aggressiveness and mycotoxin profile of Fusarium avenaceum isolates causing Fusarium seedling blight and Fusarium head blight in UK malting barley
Source: Front Plant Sci. 2023 Mar 8;14:1121553. doi: 10.3389/fpls.2023.1121553 (PMC10031139; doi:10.3389/fpls.2023.1121553)
Supplement: Supplementary file 1 [file Table_1.docx]

Supplementary Material

Aggressiveness and mycotoxin profile of *Fusarium avenaceum* isolates causing Fusarium seedling blight and Fusarium head blight in UK malting barley

Safieddin Inbaia^1^, Arifa Farooqi^2^, Rumiana V. Ray^2*^

*** Correspondence:** Rumiana V Ray: rumiana.ray@nottingham.ac.uk

| Supplementary Table 1. Sequences of primers used for quantitative real-time PCR (Nielsen et al., 2014). | | | |
| --- | --- | --- | --- |
| Target | Primer | Sequence (5’-3’) |  |
| *F. avenaceum* | Fa fwd Fa rev | TATGTTGTCACTGTCTCACACCACC AGAGGGATGTTAGCATGATGAAG |  |
|  |  |  |  |
| *F. tricinctum* | Ftri fwd Ftri rev | TTGGTATGTTGTCACTGTCTCACACT TTGACAGAGATGTTAGCATGATGCA |  |
| *F.poae* | Fp fwd Fp rev | ACCGAATCTCAACTCCGCTTT GTCTGTCAAGCATGTTAGCACAAGT |  |
|  |  |  |  |

Supplementary Table ‎2. ESI-MS/MS parameters including precursor ion, retention time (RT), cone voltage (V), Transition (m/z) and relative collision energy (eV) for beauvericin (BEA) and enniatins (ENN) A, A1, B and B1 in positive ESI interface.

| Mycotoxins | Precursor ion | RT (min) | Cone voltage (V) | Transition (m/z) | Relative Collision energy |
| --- | --- | --- | --- | --- | --- |
| BEA | 784.4 | 2.57 | 50 | 784 244 | 25 |
| ENN B | 640.4 | 2.67 | 50 | 640 196.1 | 25 |
| ENN B1 | 654.4 | 2.74 | 50 | 654 196.1 | 30 |
| ENN A1 | 668.5 | 2.84 | 50 | 668 210.2 | 27 |
| ENN A | 682.5 | 2.92 | 50 | 682 210.3 | 27 |
